# Susceptibility of wild and domestic songbirds to Usutu virus

**DOI:** 10.1371/journal.pntd.0014213

**Published:** 2026-04-16

**Authors:** Rachel D. Persinger, Jenny Buczek, Marissa Quilici, Sarah C. Kuchinsky, Casey McLaughlin, Kendra Sewall, Dana M. Hawley, Sheryl Coutermarsh-Ott, Angela M. Bosco-Lauth, Nisha K. Duggal

**Affiliations:** 1 Department of Biomedical Sciences and Pathobiology, Virginia Tech, Blacksburg, Virginia, United States of America; 2 Center for Emerging, Zoonotic, and Arthropod-borne Pathogens, Virginia Tech, Blacksburg, Virginia, United States of America; 3 Department of Biomedical Sciences, Colorado State University, Fort Collins, Colorado, United States of America; 4 Department of Biological Sciences, Virginia Tech, Blacksburg, Virginia, United States of America; UCLM: Universidad de Castilla-La Mancha, SPAIN

## Abstract

Usutu virus (USUV) is an emerging mosquito-borne orthoflavivirus that can cause neuroinvasive disease in humans and wild birds. USUV clusters phylogenetically within the Japanese encephalitis virus serocomplex, sharing antigenic and ecological similarity with West Nile virus (WNV). USUV is maintained in an enzootic cycle primarily involving passerine birds and *Culex* spp. mosquitoes. USUV was first isolated in South Africa in 1959 and has since spread throughout Africa and Europe, causing mortality and disease in several wild bird populations, specifically the Eurasian blackbird (*Turdus merula*). To understand transmission and pathogenesis of USUV in birds, we sought to develop passerine bird models of infection using wild-caught house finches (*Haemorhous mexicanus*), wild-caught American robins (*Turdus migratorius*), domestic canaries (*Serinus canaria domestica*), and captive-bred zebra finches (*Taeniopygia guttata*). Birds were inoculated with one or two isolates of USUV and viremia was measured. House finches, American robins, and canaries were susceptible to USUV, with 100% of inoculated birds developing viremia. These avian species reach viremias that have the potential to infect *Cx. quinquefasciatus* mosquitoes. Clinical disease and histopathological evidence of disease were severe in American robins and moderate to severe in canaries, with limited disease in house finches. However, zebra finches inoculated with one isolate of USUV did not develop detectable viremia. These findings provide additional tools for studying USUV enzootic transmission and pathogenesis in passerine birds.

## Author summary

Usutu virus (USUV) is an emerging arbovirus and a close relative of West Nile virus (WNV). Like WNV, USUV is maintained in a transmission cycle involving birds, primarily songbirds, and mosquitoes, mostly of the *Culex* genus. Humans and other mammals are considered dead-end hosts. In birds and humans, USUV can cause severe neuroinvasive disease. Over the past two decades, USUV has been emerging in Europe where it has caused thousands of deaths in wild bird populations. To better understand transmission and disease of USUV in wild birds, there is a need to develop avian models of infection in the lab. In this study, we aimed to develop additional passerine bird models for studying USUV, including wild-caught house finches, wild-caught American robins, domestic canaries, and captive-bred zebra finches, all of which have been previously established models for WNV. House finches, American robins, and canaries were susceptible to USUV with 100% of birds having infectious virus in the blood. Zebra finches did not become infected with USUV. The findings from these experiments provide additional passerine bird models of infection that can be used to improve our understanding of USUV transmission and disease.

## Introduction

Usutu virus (USUV, *Flaviviridae*) is an emerging mosquito-borne virus that primarily uses *Culex* spp. mosquitoes as its vector and passerine birds as its reservoir host [1–3]. USUV is a member of the Japanese encephalitis virus serocomplex, sharing similar antigenic properties and enzootic cycles in birds and mosquitoes with numerous viruses, such as West Nile virus (WNV) [4,5]. In birds, USUV can cause multisystemic and neuroinvasive disease. Humans and other mammals are considered incidental, or dead-end, hosts. In humans, USUV infection most commonly results in asymptomatic or mild febrile illness but can also lead to more severe neuroinvasive disease such as meningitis and encephalitis. To date, over 200 human cases of USUV have been documented, with the majority in Europe [6].

USUV was first isolated in South Africa in 1959 from a *Cx. neavei* mosquito [7] and then spread to Europe where it continues to circulate [8,9]. USUV has been introduced to Europe from Africa at least three times, likely by migratory birds, and then spread intracontinentally by resident bird species [3,10]. Initially, it was presumed that USUV emerged in Austria in 2001 at which time it was isolated from a dead Eurasian blackbird (*Turdus merula*) after a significant die-off event [11]. However, analysis of archived tissues from dead birds in Italy suggested USUV emerged almost five years earlier [12]. Since its emergence into Europe, USUV has caused devastating disease within bird populations, most notably the Eurasian blackbird. Over the past two decades, > 400,000 blackbirds reportedly died from USUV across Europe [13–16]. USUV also causes significant disease in several other species, including the great grey owl (*Strix nebulosa*) [11,17] and house sparrow (*Passer domesticus*) [16,18].

Thus far, USUV has been studied experimentally in several non-passerine models, including domestic geese (*Anser anser f. domestica*), red-legged partridges (*Alectoris rufa*), and domestic chickens (*Gallus gallus domesticus*). Viral RNA, but not viremia, was detected in domestic geese [19,20]. Red-legged partridges developed viremia, although at levels low enough to suggest this species is not a competent host, meaning they are unable to transmit the virus [21]. A recent study involving domestic chickens showed that juvenile chickens were susceptible to USUV and able to transmit to *Cx. quinquefasciatus* mosquitoes at low levels [22].

USUV susceptibility has also been studied in several passerine bird models of infection, including Eurasian blackbirds [23], Eurasian magpies (*Pica pica*) [24], domestic canaries (*Serinus canaria domestica*) [25], and house sparrows [26]. Eurasian magpies did not develop viremia, nor was viral RNA detectable [24]. Viral RNA was detected in Eurasian blackbirds and canaries; however, viremia was not measured [25]. House sparrows were competent for USUV, developing viremias sufficient for transmission to *Cx. quinquefasciatus* mosquitoes [26].

Given the increasing prevalence of USUV in passerines across Europe, it is crucial that we further develop passerine models to better understand susceptibility and transmission in birds. The objective of this study was to develop additional passerine bird models of USUV infection for use in studying enzootic transmission and pathogenesis. Wild-caught house finches (*Haemorhous mexicanus*), wild-caught American robins (*Turdus migratorius*), domestic canaries, and captive-bred zebra finches (*Taeniopygia guttata*), all known to be susceptible to WNV [27–30], were inoculated with USUV to evaluate their susceptibility. This study provides valuable *in vivo* passerine bird models that can be used to assess USUV transmission dynamics and disease in birds.

## Methods

### Ethics statement

All animal experiments were performed in accordance with the Virginia Tech Institutional Animal Care and Use Committee (IACUC) (approval #21–048, 23–161, and 20–186), Colorado State University IACUC (approval #6073), and federal scientific collection permits (PER0021466 and 3374565).

### Viruses and cells

USUV isolates TMNetherlands 2016 (Netherlands 2016, Africa 3 lineage, GenBank MN813490, passaged five times in Vero cells, isolated from *Turdus merula*, European Virus Archive) [17] and UG09615 (Uganda 2012, Europe 5 lineage, GenBank MN813491, passaged three times in Vero cells, isolated from *Culex univittatus*, CDC) [31], both previously sequenced by our lab [32], were used throughout these studies. Additionally, WNV NY99ic was used for plaque reduction neutralization test (PRNT) assays [33].

Vero cells were maintained at 37°C with 5% CO_2_ and were cultured in Dulbecco’s Modified Eagle Medium (DMEM, Corning) supplemented with 5% fetal bovine serum (FBS, VWR International) and 1% penicillin-streptomycin (Gibco).

### Plaque assays

Serum and tissue samples were titrated through Vero cell plaque assay. Tissues were weighed and BA-1 diluent media [34] was added to samples. Tissues were homogenized by bead homogenization at 30–50 oscillations/second for 2–6 minutes using a Qiagen TissueLyserLT and clarified by centrifugation at 18,500 rpm for 3 minutes. Serum and tissue samples were serially diluted 1:10 in BA-1 media. Using confluent Vero cells, 200 µL of each dilution was added to its respective well. Inoculated plates were incubated at 37ºC, 5% CO_2_ for 1 hour with gentle rocking every 15 minutes. Immediately after incubation, an 0.8% agarose overlay containing Ye-Lah overlay media and 3% sodium bicarbonate (Gibco) was slowly added to the wells. Two days later, a second overlay, containing 4% neutral red (Millipore Sigma), was added and plaques were counted the next day. The limits of detection were 2.0 log_10_ PFU/mL for serum samples, and 1.7 or 2.0 log_10_ PFU/g for tissue samples.

### Real-time RT-PCR

Serum was tested for viral RNA by real time quantitative reverse transcription PCR (RT-qPCR). To generate an RNA standard curve, a plasmid containing USUV NS5 [35] was linearized and *in vitro* transcribed (Ampliscribe T7 kit). The RNA was quantified and serially diluted 1:10 to create an RNA standard for use as a positive control. RT-qPCR was performed using the iTaq Universal Probes One-Step Kit (Bio-Rad Laboratories) and USUV primers (forward: 5’ CAAAGCTGGACAGACATCCCTTAC 3’, reverse: 5’ CGTAGATGTTTTCAGCCCACGT 3’) and probe (5’ FAM-AAGACATATGGTGTGGAAGCCTGATAGGCA 3’) targeting a conserved region of USUV NS5 as described previously [36]. Serum was diluted 1:10 in nuclease-free water and 4 µL of each sample was added to the RT-qPCR reaction for a total volume of 20 µL. Cycling parameters were as follows: 50°C for 30 minutes, 95°C for 15 minutes, and 40 cycles of 95°C for 15 seconds and 60°C for 1 minute (Bio-Rad CFX Connect Real-Time System). The limit of detection was 10 RNA copies/sample.

### Plaque reduction neutralization test

Blood samples collected prior to inoculation and final blood samples from birds that did not become viremic were tested by PRNT. Samples were diluted and heat-inactivated at 56°C for 30 minutes. Heat-inactivated samples were then incubated with ~100 PFU of WNV or the homologous USUV isolate at 37°C for 1 hour followed by Vero cell plaque assay. Neutralization was defined as a reduction in plaque formation by at least 90% [37].

### House finch experiments

House finches (*Haemorhous mexicanus*) of mixed sex and age were mist-netted in Blacksburg, VA during 2022 and 2023 and housed in flight cages (dimensions: 30”L x 18”W x 18”H) containing 3–4 individuals. An initial blood sample was collected by jugular or brachial wing venipuncture prior to inoculation to assess for previous WNV exposure by PRNT. Birds were divided into two groups and subcutaneously (s.c.) inoculated with 1500 PFU of the appropriate USUV isolate. A blood sample was collected daily for six days post-inoculation. Birds were monitored daily for clinical signs of disease. A final blood sample was collected prior to euthanasia, at 8 or 14 days post-inoculation (DPI), or when clinical signs, such as lethargy, puffy feathers, and lack of responsiveness, were observed. Serum was separated from whole blood by centrifugation and samples were stored at –80°C until further testing.

### Canary experiments

Adult domestic canaries (*Serinus canaria forma domestica*) of mixed sex and age were purchased from commercial vendors and housed in flight cages containing 3–4 individuals. An initial blood sample was collected prior to inoculation to assess for previous WNV exposure by PRNT. Birds were divided into two groups and s.c. inoculated with 1500 PFU of the appropriate USUV isolate. A blood sample was collected daily for six days post-inoculation. Birds were monitored daily for clinical signs of disease. A final blood sample was collected prior to euthanasia on 13 DPI, or when clinical signs were observed. Serum was separated from whole blood by centrifugation and samples were stored at –80°C until further testing. Tissues (brain, heart, liver) were collected for viral titration from a subset of canaries at time of death due to clinical signs of disease. Tissues were stored at –80°C until titration by plaque assay.

### American robin experiments

American robins (*Turdus migratorius*) of mixed sex and age were mist-netted in Fort Collins, CO during 2025 and housed in screened pop-up tents (6’L x 6’W x 6’H) containing 4 individuals. An initial blood sample was collected prior to inoculation to assess for previous WNV exposure by PRNT. Birds were s.c. inoculated with 1500 PFU of USUV isolate Netherlands 2016. A blood sample was collected daily for up to five days post-inoculation, at which time all individuals were euthanized due to clinical disease. Tissues were collected for virus isolation and histopathology at the time of euthanasia.

### Zebra finch experiments

Adult zebra finches (*Taeniopygia guttata*) of mixed sex and age were bred in-house at Virginia Tech and housed in flight cages containing 3–4 individuals. Zebra finches received cuttlebone for beak conditioning and calcium, and crushed oyster shell digestive grit*.* Birds were s.c. inoculated with 1500 PFU of USUV isolate Netherlands 2016. A blood sample was collected on 2, 4, and 6 DPI. Birds were monitored daily for clinical signs of disease. A final blood sample was collected prior to euthanasia at 14 DPI. Serum was separated from whole blood by centrifugation and samples were stored at –80°C until further testing.

### Histopathology

Tissues were collected from a subset of house finches at time of euthanasia on 8 DPI and American robins at time of euthanasia. Tissues were stored in 10% neutral buffered formalin and then, 3 days later, were replaced with 70% ethanol until further processing and paraffin embedding. Tissue sections were cut at 5 µm and stained with hematoxylin and eosin for histopathological analysis by a board-certified veterinary pathologist.

### Reservoir competence model

To model the reservoir competence index for house finches, American robins, and canaries, linear regression equations for USUV house sparrow viremia titer and *Cx. quinquefasciatus* mosquito infection rate, previously generated by our lab [26], were used for each isolate. The equation y=9.295x−22.06  was used for the Netherlands 2016 group and y=12.44x−39.77  was used for the Uganda 2012 group [26]. The linear regression equations were applied to the log-transformed viremias for each individual bird to obtain the estimated daily infectiousness, i.e., the proportion of *Cx. quinquefasciatus* mosquitoes expected to become infected after feeding on each house finch, American robin, or canary. The mean daily infectiousness was then calculated by averaging the values across all birds for each DPI. The mean daily infectiousness values were then summed to obtain the reservoir competence index. Analyses were adopted from methods described by Kilpatrick et al. for WNV [38].

### Statistical analysis

All data was analyzed and graphed using GraphPad Prism 10 (GraphPad Software, San Diego, CA). Viremia data was analyzed using two-way ANOVA with Šidák’s multiple comparisons test. Confidence intervals (CI) were calculated using the Wald method.

## Results

### House finches are susceptible to USUV

**Fig 1:**
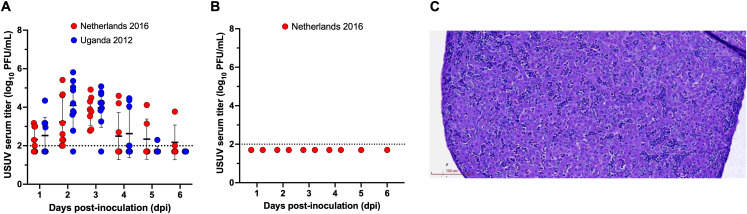
WNV seronegative house finches are susceptible to two USUV isolates. **(A)** USUV serum titer (log_10_ PFU/mL) for WNV seronegative house finches inoculated with Netherlands 2016 (*n* = 9) or Uganda 2012 (*n* = 10). **(B)** USUV viremia for two WNV-seropositive house finches. Circles represent individual birds for each DPI; horizontal lines represent the mean; error bars represent standard deviation; dashed line represents the limit of detection (LOD). Negative samples are graphed at half the LOD (1.7 log_10_ PFU/mL). **(C)** Section of liver tissue (hematoxylin and eosin [H&E] stain) from USUV Netherlands 2016-infected house finch showing lymphocytes and plasma cells (blue dots) within sinusoids and forming small aggregates in the parenchyma. Scale bar = 100 µM.

We first established a wild passerine model of USUV infection. WNV seronegative house finches were divided into two groups and s.c. inoculated with USUV Netherlands 2016 (*n* = 9) or Uganda 2012 (*n* = 10). For both groups, 100% of house finches became viremic (Netherlands 2016: 95% CI: 65.5-100%; Uganda 2012: 95% CI: 67.9-100%) with no significant differences in viremia between USUV isolates. The mean peak titer for the Netherlands 2016 group occurred at 3 DPI and was 3.8 log_10_ PFU/mL (Fig 1A). The mean peak titer for the Uganda 2012 group occurred at 2 DPI and was 4.1 log_10_ PFU/mL. Two house finches found to be naturally seropositive for WNV were also inoculated with USUV but did not become viremic and were excluded from the study (Fig 1B). No clinical signs of disease were observed in any house finches. Together, this data demonstrates that WNV-naïve house finches are susceptible to USUV.

To understand the potential for house finches to transmit USUV to mosquitoes, the reservoir competence index [39] was calculated using linear regression equations previously generated by our lab through analysis of USUV viremia titers and *Cx. quinquefasciatus* infection rates [26]. Using these equations as a model for house finches, the overall reservoir competence index was calculated to be 0.36 for Netherlands 2016 and 0.31 for Uganda 2012. These values, which are greater than zero, suggest that *Cx. quinquefasciatus* mosquitoes have the potential to become infected after feeding on USUV-infected house finches.

Histopathological analysis of tissues collected from house finches at 8 DPI were evaluated for evidence of microscopic tissue damage due to USUV infection. Lymphocytes and plasma cells were observed within sinusoids and as small aggregates in the parenchyma for the liver of a Netherlands 2016-infected house finch collected on 8 DPI (Fig 1C). Thus, house finches develop mild histopathological disease associated with USUV viral infection.

### Canaries are susceptible to USUV

**Fig 2:**
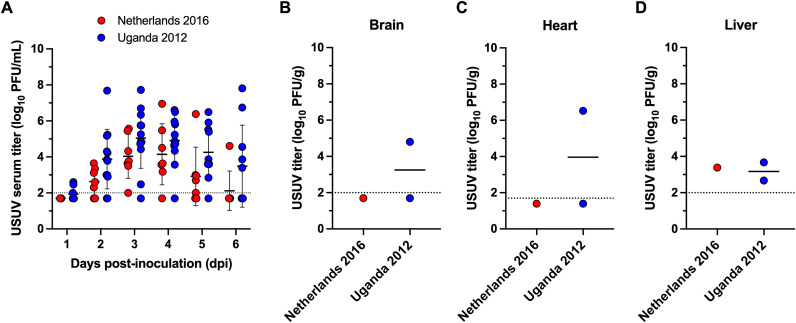
Canaries are susceptible to two USUV isolates. **(A)** USUV serum titer (log_10_ PFU/mL) for canaries inoculated with Netherlands 2016 (*n* = 7) or Uganda 2012 (*n* = 12). **(B-D)** Viral titer in brain, heart, and liver tissues, respectively. Circles represent individual birds for each DPI; horizontal lines represent the mean; dashed line represents the limit of detection (LOD). Negative samples are graphed at half the LOD (serum, brain, and liver: 1.7 log_10_ PFU/mL, heart: 1.4 log_10_ PFU/mL).

We next established a passerine model of USUV infection using domestic canaries. First, two groups of canaries, seronegative for WNV, were s.c. inoculated with USUV Netherlands 2016 (*n* = 7) or Uganda 2012 (*n* = 12). For both groups, 100% of canaries became viremic (Netherlands 2016: 95% CI: 59.6-100%; Uganda 2012: 95% CI: 71.8-100%) with no significant differences in viremia between USUV isolates at any time point. The mean peak titer for the Netherlands 2016 group occurred at 4 DPI and was 4.1 log_10_ PFU/mL (Fig 2A). The mean peak titer for the Uganda 2012 group occurred at 3 DPI and was 5.0 log_10_ PFU/mL.

To understand the potential for canaries to transmit USUV to mosquitoes, the reservoir competence index was calculated using the model previously described above. The overall reservoir competence index was 0.40 for Netherlands 2016 and 0.89 for Uganda 2012. These values suggest that *Cx. quinquefasciatus* mosquitoes may become infected after feeding on USUV-infected canaries.

Clinical signs of disease were observed between 4–8 DPI in five individuals. Of those, three individuals met humane euthanasia criteria, and tissues were collected to assess viral dissemination. Infectious virus was isolated from the brain and heart tissues for one Uganda 2012-infected canary (Fig 2B and 2C). Infectious virus was also isolated from the liver of one Netherlands 2016- and two Uganda 2012-infected individuals (Fig 2D). These results indicate that canaries are susceptible to USUV and develop moderate to severe clinical disease following virus dissemination.

### Severe disease in USUV-inoculated American robins

**Fig 3:**
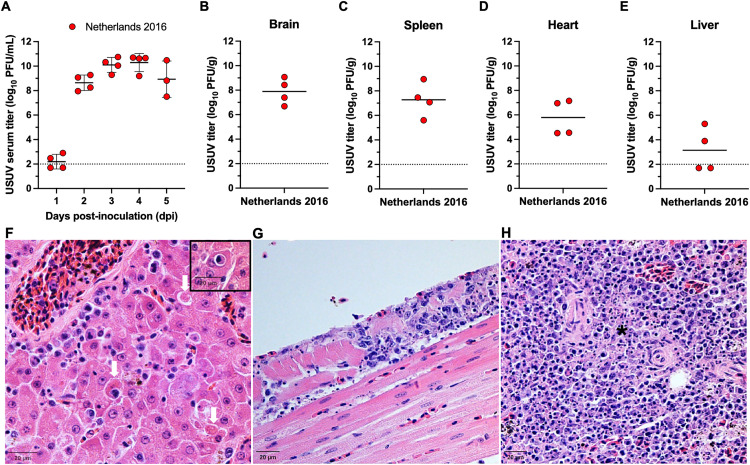
American robins are susceptible to USUV Netherlands 2016. **(A)** USUV serum titer (log_10_ PFU/mL) for American robins inoculated with Netherlands 2016 (*n* = 4). **(B-E)** Viral titer in brain, spleen, heart, and liver tissues, respectively. Circles represent individual birds for each DPI; horizontal lines represent the mean; error bars represent standard deviation; dashed line represents the limit of detection (LOD). Negative samples are graphed at half the LOD (1.7 log_10_ PFU/mL). **(F)** Section of liver tissue (hematoxylin and eosin [H&E] stain) from a USUV Netherlands 2016-infected robin showing individual hepatocellular necrosis (white arrows) with a mild mononuclear inflammatory infiltrate **(inset image)**. Rarely, hepatocytes demonstrated lymphocyte emperipolesis. **(G)** Section of heart tissue (H&E stain) from a USUV Netherlands 2016-infected robin showing mild multifocal lymphohistiocytic endocarditis affecting the right ventricular endocardium. **(H)** Section of splenic tissue (H&E stain) from a USUV Netherlands 2016-infected robin showing marked multifocal splenic lymphoid necrosis (*). Scale bars = 20 µM.

We next investigated the susceptibility of American robins to USUV. One group of American robins was s.c. inoculated with USUV isolate Netherlands 2016 (*n* = 4). 100% of American robins became viremic (95% CI: 45.4-100%) with a mean peak titer of 10.3 log_10_ PFU/mL on 4 DPI (Fig 3A). Using the previously described equations as a model, the overall reservoir competence index was calculated to be 2.66, which suggests that all *Cx. quinquefasciatus* mosquitoes may become infected after feeding on a Netherlands 2016-infected American robin.

All American robins developed severe clinical disease and were euthanized between 5–6 DPI. Infectious virus was isolated from the brain, heart, and spleen of all birds, and from the liver of 2 of the birds (Fig 3B–3E). On histologic examination, the most common findings included mild (*n* = 1) to moderate (*n* = 2) to marked (*n* = 1) lymphohistiocytic hepatitis in 4/4 birds, splenic lymphoid necrosis and myocardial lesions in 3/4 birds, and moderate (*n* = 2) to marked (*n* = 2) lymphoplasmacytic ileotyphlitis and enteritis in 4/4 birds (Fig 3F–3H). These results indicate that American robins are susceptible to USUV and develop severe clinical disease following virus dissemination.

### Zebra finches are not susceptible to a European USUV isolate

**Fig 4:**
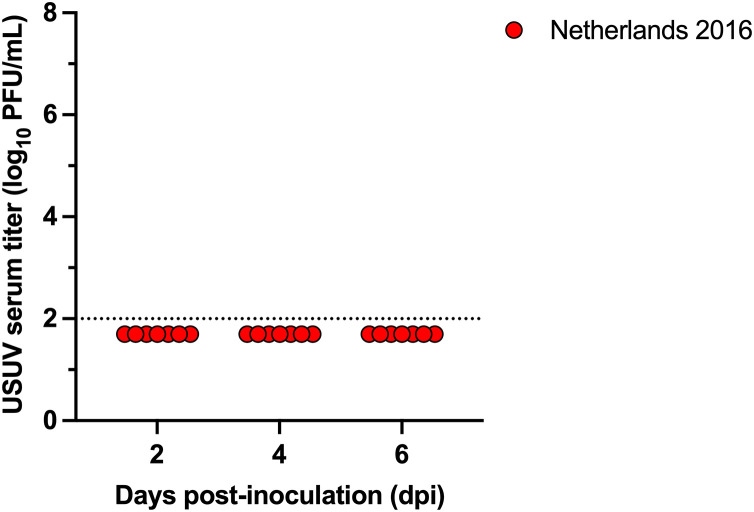
Zebra finches are not susceptible to USUV Netherlands 2016. USUV serum titer (log_10_ PFU/mL) for zebra finches inoculated with Netherlands 2016 (*n* = 7) Circles represent individual birds for each DPI; dashed line represents the limit of detection (LOD). Negative samples are graphed at half the LOD (1.7 log_10_ PFU/mL).

Next, zebra finches (*n* = 7) were s.c. inoculated with USUV isolate Netherlands 2016. Zero percent of zebra finches became viremic (95% CI: 0-40.4%) at 2, 4, and 6 DPI (Fig 4). Additionally, RT-qPCR was performed on serum from 2 DPI, and viral RNA was not detected. A final blood sample was collected at 14 DPI to test for seroconversion by PRNT; no individuals developed neutralizing antibodies against USUV. This data suggests that at a dose of 1500 PFU and using the sampling scheme used, zebra finches do not develop a systemic infection to USUV isolate Netherlands 2016.

## Discussion

In these studies, we found that house finches, American robins, and domestic canaries are susceptible to USUV. Viremia from individuals of each of these species reached levels that, based on our models, may be sufficient to infect *Cx. quinquefasciatus* mosquitoes. In a subset of USUV-infected canaries, virus disseminated to several tissues and caused moderate to severe clinical disease. In American robins, USUV infection caused severe clinical disease and high levels of virus dissemination in all birds. However, zebra finches did not become viremic when inoculated with USUV.

American robins are in the same genus as the Eurasian blackbird, which are frequently found dead in the wild from USUV infection in Europe [13–15]. Recent work has demonstrated that experimental USUV infection in Eurasian blackbirds leads to severe disease, with 100% mortality between 4–6 DPI [23]. Here, using a small group size, we found that American robins are also highly susceptible to USUV, with severe clinical disease and 100% mortality between 5–6 DPI. Histopathologic lesions common to both species include splenomegaly, hepatic degeneration and necrosis, and mild-to-absent CNS lesions. However, in American robins, myocarditis and enteritis were also observed, whereas blackbirds were more likely to develop lesions consistent with interstitial pneumonia. It cannot be ruled out that these differences between American robins and Eurasian blackbirds may be due to differences in the USUV isolates or experimental settings used between the studies. Additionally, we did not include sham-inoculated birds to limit our impact on the wild bird population; however, sham-inoculated birds would be a valuable control for the effects of being held in captivity. Nevertheless, while subtle differences in tissue tropism and systemic pathogenesis may exist across highly susceptible species, American robins could be a model for severe USUV disease in birds, although additional experimentation, such as increasing group sizes and including a sham-inoculated group, is warranted to fully capture USUV disease in this species.

Histopathologic analysis of house finch tissues showed mild microscopic tissue damage related to USUV disease in the liver. This mainly consisted of inflammation and immune cell infiltrates which is consistent with results for the liver of house sparrows experimentally infected with USUV [26]. Together, these histopathological findings align with studies of naturally infected wild birds across Europe [40–43]. Interestingly, despite high viremia levels and histopathological findings in the liver, no clinical signs were observed in USUV-infected house finches prior to euthanasia. While further study is needed, including incorporating a sham-inoculated control group and assessment of viral titers in tissues, both limitations of our study, house finches infected with USUV may not experience the degree of mortality seen in some wild bird populations in Europe. If true, this may allow house finches to serve as reservoirs of USUV.

In our study, two house finches were found to be positive for WNV antibodies prior to USUV inoculation. Interestingly, these two individuals did not develop USUV viremia. In a previous study, house sparrows experimentally infected with WNV were completely protected against subsequent flavivirus infection, including USUV Netherlands 2016, for at least one month [44]. Further studies evaluating the duration of cross-protective immunity in passerine birds using larger group sizes and controlled challenge experiments are warranted. Nonetheless, the house finch model developed in this study can be used as a tool to investigate flavivirus cross-protective immune responses in birds.

Using previously derived linear regression equations from our lab as a model [26], the reservoir competence index calculated for house finches and American robins in our study suggests that these bird species have the potential to transmit USUV to North American *Cx. quinquefasciatus* mosquitoes, a species that is widely distributed across the Americas [45]. When compared to the reservoir competence indexes previously calculated for house sparrows (average of 0.22 [26]), the reservoir competence of house finches (average of 0.34) is similar and suggests moderate competence for USUV transmission. American robins, on the other hand, are expected to infect a much greater number of mosquitoes than house sparrows or house finches (reservoir competence of 2.66), suggesting they could be a major host for transmitting USUV to *Cx. quinquefasciatus*. In fact, the reservoir competence of American robins for USUV is similar to the most competent songbirds for WNV transmission, including American crows and blue jays [27]. It is important to consider that the estimates of reservoir competence we calculated in our study are derived from a model using a single mosquito species where mosquitoes were reared under laboratory conditions, which may not fully capture real-world transmission dynamics that can be affected by other variables, such as feeding behavior and environmental factors. However, future studies evaluating transmission dynamics between *Culex* spp. mosquitoes and these bird species using empirical studies would be important, as previous research has shown variability in *Cx. pipiens* infection rates between different bird species infected with WNV, even when viremia levels are the same [46].

To date, there are few domestic passerine bird models to study USUV, and our data aligns with previous work establishing canaries as a potential model system. A previous study investigating the susceptibility of canaries inoculated with 10^3^ TCID_50_ of USUV isolate Italy 2010 found approximately 6 log_10_ viral RNA copies in the serum at 3 DPI [25]. Considering that viral RNA levels are generally higher than infectious virus titers, this data is consistent with the amount of infectious virus we observed at 3 DPI (4–5 log_10_ PFU/mL) (Fig 2). In addition, we observed moderate to severe clinical signs of disease and virus dissemination to tissues, similar to reports in wild birds [42,43]. Therefore, canaries are an appropriate domestic bird model for studying moderate to severe USUV pathogenesis.

Our study demonstrated that zebra finches did not develop a systemic infection to USUV isolate Netherlands 2016 at a dose of 1500 PFU under an infrequent sampling scheme, a limitation of our study design. It is unclear if zebra finches are resistant to USUV or if they may require a higher dose for infection, though previous studies have found that increasing the USUV dose did not increase viremia in chickens [22]. In contrast, house finches reached peak viremias of 4 log_10_ PFU/mL, canaries reached peak viremias of 4–5 log_10_ PFU/mL of USUV, and American robins reached peak viremias of 10 log_10_ PFU/mL of USUV. While all four species are passerines, house finches and canaries belong to the *Fringillidae* family whereas American robins belong to the *Turdidae* family, and zebra finches belong to the *Estrildidae* family. We do note that differences in the study design between species limit our ability to directly compare results across these species. However, these results suggest that susceptibility of USUV infection may be species or family specific within the passerine order. This is consistent across other flaviviruses. For example, zebra finches s.c. inoculated with 10^3^ PFU of WNV NY99 reached peak viremias of approximately 5 log_10_ PFU/mL [30]. In contrast, house finches, s.c. inoculated with 10^3^ PFU of WNV NY99, and canaries s.c. inoculated with 10^1^ PFU of WNV NY99, reached peak viremias of approximately 7 and 10 log_10_ PFU/mL, respectively [28,29]. American robins s.c. inoculated with 10^3^ PFU of WNV NY99 reached peak viremias of approximately 6 log_10_ PFU/mL [47]. Thus, for both WNV and USUV, the mean peak viremias for zebra finches are consistently lower than other passerine species (Table 1). In addition, while the mean peak viremia for USUV is several orders of magnitude lower than WNV for birds in the *Fringillidae* family, the peak viremia for USUV is several orders of magnitude higher than WNV in American robins, which are in the *Turdidae* family. Together, this suggests that WNV and USUV may be differentially adapted to specific passerine families. Future experimentation is needed to determine if higher doses are necessary to develop USUV viremia in zebra finches and to understand the differential susceptibility of passerine species to WNV and USUV.

**Table 1:** Approximate mean peak viremias for songbirds inoculated with WNV (published studies) or USUV (this study).

	Zebra finches^*^	House finches^*^	Canaries^*^	American robins^*^
**WNV**	5^†^ [30]	7^†^ [28]	10^†^ [29]	6^†^ [47]
**USUV**	<2	4	4-5	10

* Subcutaneously inoculated.

† Values are approximate. Values are reported as log_10_ PFU/mL.

USUV is an emerging virus that has had a devastating impact on wild bird populations in Europe, primarily passerine species, over the past two decades [13–16]. Here, we describe several passerine models of infection for USUV, both wild and domestic. This work could contribute to our understanding of the ecology and epidemiology of USUV and could help to identify factors that influence USUV transmission. Altogether, the tools developed in these studies will be vital in further understanding USUV pathogenesis in birds and enzootic transmission dynamics.

## Supporting information

S1 Data Data from Figures 1–4. (XLSX)

## References

[1] Gaibani P, Rossini G. An overview of Usutu virus. Microbes Infect. 2017;19(7–8):382–7. doi: 10.1016/j.micinf.2017.05.003 28602915

[2] Vazquez A, Jimenez-Clavero M, Franco L, Donoso-Mantke O, Sambri V, Niedrig M, et al. Usutu virus: potential risk of human disease in Europe. Eurosurveillance. 2011;16(31):19935. 21871214

[3] Clé M, Beck C, Salinas S, Lecollinet S, Gutierrez S, Van de Perre P, et al. Usutu virus: a new threat? Epidemiol Infect. 2019;147:e232. doi: 10.1017/s0950268819001213 31364580 PMC6625183

[4] Khare B, Kuhn RJ. The Japanese encephalitis antigenic complex viruses: from structure to immunity. Viruses. 2022;14(10):2213. doi: 10.3390/v14102213 36298768 PMC9607441

[5] Roesch F, Fajardo A, Moratorio G, Vignuzzi M. Usutu virus: an arbovirus on the rise. Viruses. 2019;11(7):640. doi: 10.3390/v11070640 31336826 PMC6669749

[6] Cadar D, Simonin Y. Human usutu virus infections in Europe: a new risk on horizon? Viruses. 2022;15(1):Epub 20221227. doi: 10.3390/v15010077 36680117 PMC9866956

[7] Williams MC, Simpson DI, Haddow AJ, Knight EM. The isolation of West Nile virus from man and of Usutu virus from the bird-biting mosquito Mansonia Aurites (Theobald) in the Entebbe area of Uganda. Ann Trop Med Parasitol. 1964;58:367–74. doi: 10.1080/00034983.1964.11686258 14212897

[8] Vilibic-Cavlek T, Petrovic T, Savic V, Barbic L, Tabain I, Stevanovic V. Epidemiology of Usutu virus: the European scenario. Pathogens. 2020;9(9):699. doi: 10.3390/pathogens9090699 32858963 PMC7560012

[9] Simonin Y. Circulation of West Nile virus and Usutu virus in Europe: overview and challenges. Viruses. 2024;16(4):599. doi: 10.3390/v16040599 38675940 PMC11055060

[10] Engel D, Jöst H, Wink M, Börstler J, Bosch S, Garigliany M-M, et al. Reconstruction of the evolutionary history and dispersal of Usutu virus, a neglected emerging arbovirus in Europe and Africa. mBio. 2016;7(1):e01938-15. doi: 10.1128/mBio.01938-15 26838717 PMC4742707

[11] Weissenböck H, Kolodziejek J, Url A, Lussy H, Rebel-Bauder B, Nowotny N. Emergence of Usutu virus, an African mosquito-borne flavivirus of the Japanese encephalitis virus group, central Europe. Emerg Infect Dis. 2002;8(7):652–6. doi: 10.3201/eid0807.020094 12095429 PMC2730324

[12] Weissenböck H, Bakonyi T, Rossi G, Mani P, Nowotny N. Usutu virus, Italy, 1996. Emerg Infect Dis. 2013;19(2):274–7. doi: 10.3201/eid1902.121191 23347844 PMC3559058

[13] Chvala S, Bakonyi T, Bukovsky C, Meister T, Brugger K, Rubel F. Monitoring of Usutu virus activity and spread by using dead bird surveillance in Austria, 2003–2005. Vet Microbiol. 2007;122(3):237–45. doi: 10.1016/j.vetmic.2007.01.029 17346908

[14] Savini G, Monaco F, Terregino C, Di Gennaro A, Bano L, Pinoni C, et al. Usutu virus in Italy: an emergence or a silent infection? Vet Microbiol. 2011;151(3):264–74. doi: 10.1016/j.vetmic.2011.03.036 21550731

[15] Lühken R, Jöst H, Cadar D, Thomas SM, Bosch S, Tannich E, et al. Distribution of Usutu virus in Germany and its effect on breeding bird populations. Emerg Infect Dis. 2017;23(12):1994–2001. doi: 10.3201/eid2312.171257 29148399 PMC5708248

[16] Folly AJ, Lawson B, Lean FZ, McCracken F, Spiro S, John SK, et al. Detection of Usutu virus infection in wild birds in the United Kingdom, 2020. Eurosurveillance. 2020;25(41):2001732. doi: 10.2807/1560-7917.ES.2020.25.41.2001732 33063656 PMC7565854

[17] Rijks JM, Kik ML, Slaterus R, Foppen R, Stroo A, IJzer J, et al. Widespread Usutu virus outbreak in birds in the Netherlands, 2016. Eurosurveillance. 2016;21(45):30391. doi: 10.2807/1560-7917.ES.2016.21.45.30391 27918257 PMC5144937

[18] Steinmetz HW, Bakonyi T, Weissenböck H, Hatt J-M, Eulenberger U, Robert N, et al. Emergence and establishment of Usutu virus infection in wild and captive avian species in and around Zurich, Switzerland--Genomic and pathologic comparison to other central European outbreaks. Vet Microbiol. 2011;148(2–4):207–12. doi: 10.1016/j.vetmic.2010.09.018 20980109

[19] Chvala S, Bakonyi T, Hackl R, Hess M, Nowotny N, Weissenböck H. Limited pathogenicity of usutu virus for the domestic goose (Anser anser f. domestica) following experimental inoculation. J Vet Med B Infect Dis Vet Public Health. 2006;53(4):171–5. doi: 10.1111/j.1439-0450.2006.00942.x 16629984

[20] Reemtsma H, Holicki CM, Fast C, Bergmann F, Groschup MH, Ziegler U. A Prior Usutu virus infection can protect geese from severe West Nile disease. Pathogens. 2023;12(7):959. doi: 10.3390/pathogens12070959 37513806 PMC10386565

[21] Llorente F, Gutiérrez-López R, Pérez-Ramirez E, Sánchez-Seco MP, Herrero L, Jiménez-Clavero MÁ, et al. Experimental infections in red-legged partridges reveal differences in host competence between West Nile and Usutu virus strains from Southern Spain. Front Cell Infect Microbiol. 2023;13:1163467. doi: 10.3389/fcimb.2023.1163467 37396301 PMC10308050

[22] Kuchinsky SC, Frere F, Heitzman-Breen N, Golden J, Vázquez A, Honaker CF, et al. Pathogenesis and shedding of Usutu virus in juvenile chickens. Emerg Microbes Infect. 2021;10(1):725–38. doi: 10.1080/22221751.2021.1908850 33769213 PMC8043533

[23] Agliani G, Visser I, Marshall EM, Giglia G, de Bruin E, Verstappen R, et al. Experimental Usutu virus infection in Eurasian blackbirds (Turdus merula). Npj Viruses. 2025;3(1):51. doi: 10.1038/s44298-025-00133-w 40542200 PMC12181337

[24] Escribano-Romero E, Jiménez de Oya N, Camacho M-C, Blázquez A-B, Martín-Acebes MA, Risalde MA, et al. Previous Usutu virus exposure partially protects magpies (Pica pica) against West Nile virus disease but does not prevent horizontal transmission. Viruses. 2021;13(7):1409. doi: 10.3390/v13071409 34372622 PMC8310384

[25] Benzarti E, Rivas J, Sarlet M, Franssen M, Desmecht D, Schmidt-Chanasit J, et al. Experimental Usutu virus infection in domestic canaries Serinus canaria. Viruses. 2020;12(2):164. doi: 10.3390/v12020164 32023880 PMC7077186

[26] Kuchinsky SC, Marano J, Hawks SA, Loessberg E, Honaker CF, Siegel PB, et al. North American house sparrows are competent for Usutu virus transmission. mSphere. 2022;7(6):e0029522. doi: 10.1128/msphere.00295-22 36317895 PMC9769741

[27] Komar N, Langevin S, Hinten S, Nemeth N, Edwards E, Hettler D, et al. Experimental infection of North American birds with the New York 1999 strain of West Nile virus. Emerg Infect Dis. 2003;9(3):311–22. doi: 10.3201/eid0903.020628 12643825 PMC2958552

[28] Reisen WK, Fang Y, Martinez VM. Avian host and mosquito (Diptera: Culicidae) vector competence determine the efficiency of West Nile and St. Louis encephalitis virus transmission. J Med Entomol. 2005;42(3):367–75. doi: 10.1093/jmedent/42.3.367 15962789

[29] Hofmeister EK, Lund M, Shearn Bochsler V. West Nile virus infection in American singer canaries: an experimental model in a highly susceptible avian species. Vet Pathol. 2018;55(4):531–8. doi: 10.1177/0300985818760377 29506438

[30] Hofmeister EK, Lund M, Shearn-Bochsler V, Balakrishnan CN. Susceptibility and antibody response of the laboratory model Zebra Finch (Taeniopygia guttata) to West Nile virus. PLoS One. 2017;12(1):e0167876. doi: 10.1371/journal.pone.0167876 28045891 PMC5207765

[31] Mossel EC, Crabtree MB, Mutebi JP, Lutwama JJ, Borland EM, Powers AM. Arboviruses isolated from mosquitoes collected in Uganda, 2008-2012. J Med Entomol. 2017;54(5):1403–9. doi: 10.1093/jme/tjx120 28874015 PMC5968633

[32] Kuchinsky SC, Hawks SA, Mossel EC, Coutermarsh-Ott S, Duggal NK. Differential pathogenesis of Usutu virus isolates in mice. PLoS Negl Trop Dis. 2020;14(10):e0008765. doi: 10.1371/journal.pntd.0008765 33044987 PMC7580916

[33] Kinney RM, Huang CY-H, Whiteman MC, Bowen RA, Langevin SA, Miller BR, et al. Avian virulence and thermostable replication of the North American strain of West Nile virus. J Gen Virol. 2006;87(Pt 12):3611–22. doi: 10.1099/vir.0.82299-0 17098976

[34] Salgado R, Hawks SA, Frere F, Vázquez A, Huang CY, Duggal NK. West Nile virus vaccination protects against Usutu virus disease in mice. Viruses. 2021;13(12). doi: 10.3390/v13122352 34960621 PMC8704473

[35] Persinger RD, Kuchinsky SC, Cereghino C, Murphy QM, Liyanage YR, Tuncer N, et al. North American Culex pipiens mosquitoes are competent for Usutu virus transmission. Npj Viruses. 2026;4(1):16. doi: 10.1038/s44298-026-00182-9 41787077 PMC12963485

[36] Nikolay B, Weidmann M, Dupressoir A, Faye O, Boye CS, Diallo M, et al. Development of a Usutu virus specific real-time reverse transcription PCR assay based on sequenced strains from Africa and Europe. J Virol Methods. 2014;197:51–4. doi: 10.1016/j.jviromet.2013.08.039 24036076

[37] Duggal NK, Ritter JM, Pestorius SE, Zaki SR, Davis BS, Chang G-JJ, et al. Frequent Zika virus sexual transmission and prolonged viral RNA shedding in an immunodeficient mouse model. Cell Rep. 2017;18(7):1751–60. doi: 10.1016/j.celrep.2017.01.056 28199846 PMC5683178

[38] Kilpatrick AM, LaDeau SL, Marra PP. Ecology of West Nile virus transmission and its impact on birds in the western hemisphere. The Auk. 2007;124(4):1121–36. doi: 10.1093/auk/124.4.1121

[39] Komar N, Dohm DJ, Turell MJ, Spielman A. Eastern equine encephalitis virus in birds: relative competence of European starlings (Sturnus vulgaris). Am J Trop Med Hyg. 1999;60(3):387–91. doi: 10.4269/ajtmh.1999.60.387 10466964

[40] Giglia G, Agliani G, Munnink BBO, Sikkema RS, Mandara MT, Lepri E, et al. Pathology and pathogenesis of Eurasian blackbirds (Turdus merula) naturally infected with Usutu virus. Viruses. 2021;13(8):1481. doi: 10.3390/v13081481 34452347 PMC8402641

[41] Weissenböck H, Kolodziejek J, Fragner K, Kuhn R, Pfeffer M, Nowotny N. Usutu virus activity in Austria, 2001-2002. Microbes Infect. 2003;5(12):1132–6. doi: 10.1016/s1286-4579(03)00204-1 14554255

[42] Manarolla G, Bakonyi T, Gallazzi D, Crosta L, Weissenböck H, Dorrestein GM, et al. Usutu virus in wild birds in northern Italy. Vet Microbiol. 2010;141(1–2):159–63. doi: 10.1016/j.vetmic.2009.07.036 19720475

[43] Chvala S, Kolodziejek J, Nowotny N, Weissenböck H. Pathology and viral distribution in fatal Usutu virus infections of birds from the 2001 and 2002 outbreaks in Austria. J Comp Pathol. 2004;131(2–3):176–85. doi: 10.1016/j.jcpa.2004.03.004 15276857

[44] Bosco-Lauth AM, Kooi K, Hawks SA, Duggal NK. Cross-protection between West Nile virus and emerging flaviviruses in wild birds. Am J Trop Med Hyg. 2024;112(3):657–62. doi: 10.4269/ajtmh.24-0363 39742522 PMC11884272

[45] Gorris ME, Bartlow AW, Temple SD, Romero-Alvarez D, Shutt DP, Fair JM, et al. Updated distribution maps of predominant Culex mosquitoes across the Americas. Parasit Vectors. 2021;14(1):547. doi: 10.1186/s13071-021-05051-3 34688314 PMC8542338

[46] Vaughan JA, Newman RA, Turell MJ. Bird species define the relationship between West Nile viremia and infectiousness to Culex pipiens mosquitoes. PLoS Negl Trop Dis. 2022;16(10):e0010835. doi: 10.1371/journal.pntd.0010835 36201566 PMC9578590

[47] Grubaugh ND, Smith DR, Brackney DE, Bosco-Lauth AM, Fauver JR, Campbell CL, et al. Experimental evolution of an RNA virus in wild birds: evidence for host-dependent impacts on population structure and competitive fitness. PLoS Pathog. 2015;11(5):e1004874. doi: 10.1371/journal.ppat.1004874 25993022 PMC4439088

